# Randomised Placebo-Controlled Pilot Trial Evaluating the Anti-Anginal Efficacy of Ticagrelor in Patients with Angina with Nonobstructive Coronary Arteries and Coronary Slow Flow Phenomenon

**DOI:** 10.3390/jcm13175235

**Published:** 2024-09-04

**Authors:** Sivabaskari Pasupathy, Rosanna Tavella, Christopher Zeitz, Suzanne Edwards, Matthew Worthley, Margaret Arstall, John F. Beltrame

**Affiliations:** 1 School of Medicine, Faculty of Health and Medical Sciences, The University of Adelaide, Adelaide, SA 5000, Australia; rosanna.tavella@adelaide.edu.au (R.T.); christopher.zeitz@adelaide.edu.au (C.Z.); matthew.worthley@adelaide.edu.au (M.W.); margaret.arstall@adelaide.edu.au (M.A.); john.beltrame@adelaide.edu.au (J.F.B.); 2 Central Adelaide Local Health Network, Adelaide, SA 5000, Australia; 3 Basil Hetzel Institute for Translational Health Research, Adelaide, SA 5011, Australia; 4 Flinders University, Adelaide, SA 5042, Australia; 5 School of Public Health, Faculty of Health and Medical Sciences, The University of Adelaide, Adelaide, SA 5000, Australia; suzanne.edwards@adelaide.edu.au; 6 Northern Adelaide Local Health Network, Adelaide, SA 5112, Australia

**Keywords:** coronary slow flow phenomenon, clinical trial, ticagrelor, angina

## Abstract

**Background**: The coronary slow flow phenomenon (CSFP) is an angiographic finding characterised by the delayed passage of contrast through the coronary arteries, despite the absence of obstructive coronary artery disease (defined as less than 50% narrowing of the vessel lumen). Patients with the CSFP experience recurrent angina, for which there are limited evidence-based therapies. Ticagrelor may serve as an effective anti-anginal therapy for these patients by increasing adenosine levels, which could alleviate coronary microvascular dysfunction and its associated angina due to its vasodilatory properties. This study aimed to determine the anti-anginal efficacy of ticagrelor 90 mg taken twice daily on spontaneous angina episodes in patients with refractory angina (i.e., episodes ≥3/week despite two anti-anginals) and documented CSFP. **Methods**: In a randomised, double-blind, placebo-controlled, cross-over trial, the anti-anginal efficacy of a 4-week ticagrelor therapy regimen was evaluated in 20 patients with refractory angina (mean age 61.5 ± 10.5 years; 40% women) who had documented slow coronary flow. The primary endpoint was the frequency of angina episodes, recorded using an angina diary. Secondary endpoints included the duration and severity of angina episodes, consumption of short-acting nitrates, and health status evaluations using the Seattle Angina Questionnaire (SAQ) and the Short Form-36 (SF-36) indices. **Results:** During the four weeks of therapy, ticagrelor did not significantly improve angina symptoms compared to the placebo (placebo 25.7 (16.7)) vs. ticagrelor 19.8 (18.1), *p* > 0.05). Furthermore, it did not impact other patient-related outcome measures, including angina severity, duration, frequency of prolonged angina episodes, nitrate consumption, or the SAQ/SF-36 health outcome indices. No serious adverse events related to the study drug were observed. **Conclusions**: In patients with documented CSFP who were unresponsive to standard anti-anginal therapy, ticagrelor did not reduce the frequency of spontaneous angina episodes or the consumption of nitrates. Further confirmation of the potential benefits of this therapy may be obtained through a larger clinical trial.

## 1. Introduction

Angina, a clinical manifestation of myocardial ischaemia, can be attributed to various pathologies affecting both large coronary vessels and the microvasculature. Large-vessel causes include atherosclerotic coronary artery disease and coronary artery spasm, while microvascular causes encompass microvascular spasm and coronary microvascular dysfunction (CMD). Therapies to treat epicardial coronary artery disease are well-established and include both revascularisation therapies and anti-anginal medication. Coronary artery spasm, whether microvascular or epicardial, is primarily managed with calcium channel blockers and anti-anginal agents. However, the treatment of coronary microvascular dysfunction is more challenging [1] and may require different treatments from those established for large vessel atherosclerotic coronary artery disease and coronary artery spasm. Coronary microvascular dysfunction (CMD) is an ‘abnormal coronary microvascular resistance (either arteriolar or pre-arteriolar) that is clinically evident as an inappropriate coronary blood flow response, impaired myocardial perfusion, and/or myocardial ischaemia that cannot be accounted for by abnormalities in the epicardial coronary arteries’ [2]. CMD can be assessed using both invasive and non-invasive methods. Invasive techniques include intracoronary Doppler wire and intracoronary thermodilution, which provide detailed measurements of coronary blood flow but come with higher complexity and risk [3,4]. Non-invasive methods include Cardiac Positron Emission Tomography, which measures myocardial blood flow; Cardiac Magnetic Resonance Imaging, which uses gadolinium contrast to evaluate microvascular function; Myocardial Contrast Echocardiography, which assesses myocardial blood flow using echocardiographic contrast; and Transthoracic Doppler Echocardiography, which measures blood flow in the left anterior descending coronary artery [5].

The coronary slow flow phenomenon (CSFP) is a microvascular disorder in which there is delayed contrast opacification of the distal coronary vessels during invasive coronary angiography, despite the absence of obstructive lesions in the major coronary arteries (i.e., no epicardial lesion ≥ 50%) [6]. This phenomenon reflects CMD. Diagnosis is based on TIMI flow grade or TIMI frame count. A TIMI-2 flow grade, where the vessel requires at least three beats to opacify and a corrected TIMI frame count exceeding 27 frames are commonly used criteria. The corrected TIMI frame count is derived from images captured at 30 frames per second, with a correction factor of 1.7 applied for the left anterior descending artery. Patients with CSFP typically present with symptoms like those of atherosclerotic coronary artery disease. They commonly experience angina, characterised by chest pain that may be stable or unstable and is often described as pressure or tightness, which can radiate to the arms, neck, jaw, or back. Additionally, they may suffer from dyspnoea, or shortness of breath, particularly during physical activity. Fatigue is also a frequent complaint, manifesting as generalised tiredness that is often exacerbated by exertion. These symptoms can be chronic and recurrent, significantly affecting the patient’s quality of life. The 2023 Guidelines [7] for the Management of Chronic Coronary Disease address various conditions related to coronary artery dysfunction; however, they do not include the coronary slow flow phenomenon. Consequently, there are no established guidelines for the treatment of patients with this condition. Clinically distinct from other microvascular disorders, the mechanisms responsible for the microvascular dysfunction remain elusive, although a plethora of pathophysiological investigations have reported abnormalities in endothelial function [8,9,10], inflammatory markers [11,12], oxidative stress markers [10,13], and platelets [14]. However, these patients consistently exhibit increased microvascular resistance [15,16], suggesting that coronary microvascular vasodilator therapy may be an effective treatment approach.

Ticagrelor is a well-established anti-platelet agent used in the management of large vessel coronary artery disease. This effect is mediated by inhibiting the P2Y12 platelet receptor, but ticagrelor also inhibits adenosine uptake into cells, thereby increasing adenosine levels [17]. Adenosine is a potent coronary microvascular vasodilator used to evaluate the capacity of the coronary microvasculature to vasodilate (i.e., coronary flow reserve measurement). Hence, by increasing adenosine levels, ticagrelor may alleviate coronary microvascular dysfunction and its associated angina. Studies on healthy subjects have demonstrated that ticagrelor augments adenosine-induced coronary blood flow, which is inhibited by theophylline, a non-selective adenosine receptor antagonist [18]. However, the specific coronary hyperaemic effect of ticagrelor in patients with coronary heart disease, particularly those with CMD, remains unclear.

The primary aim of this study was to evaluate whether administering ticagrelor at a dose of 90 mg twice daily would affect the frequency of angina in patients experiencing symptomatic CSFP. The secondary objectives involved an evaluation of the effects of ticagrelor therapy on other clinical outcomes, including the frequency of prolonged angina episodes, consumption of short-acting nitrates, and health status, as assessed by the Seattle Angina Questionnaire (SAQ) and Short Form-36 (SF-36) indices.

## 2. Materials and Methods

### 2.1. Study Design

**Figure 1:**
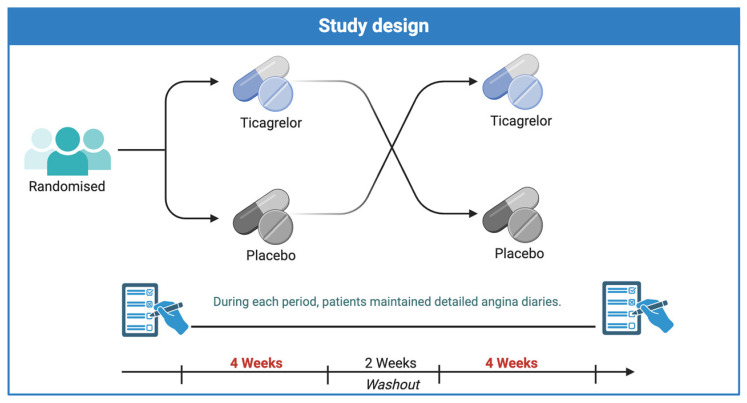
Schematic representation of the study design, depicting the sequential phases from initial patient enrolment, randomisation (green) into ticagrelor (blue) or placebo (grey) groups, the duration of each treatment phase (4 weeks), the two-week washout period, and the subsequent crossover to the alternate treatment. Created with BioRender.com.

This pilot study was a single-centre, randomised, double-blind, placebo-controlled, cross-over clinical trial aimed at evaluating the efficacy of ticagrelor over 4 weeks of treatment (Figure 1). The primary efficacy measure was angina frequency derived from an angina diary. The active medication used in the study was 90 mg ticagrelor (AstraZeneca UK Ltd., Cambridge Biomedical Campus, Cambridge, UK), administered orally twice daily. An identical placebo, indistinguishable from the active drug in appearance, was also provided by the manufacturer. The study protocol was approved by the Central Adelaide Local Health Network ethics committee, and all participants provided written informed consent prior to the commencement of any study procedures.

### 2.2. Study Population

Both recently diagnosed and established patients with CSFP were eligible for recruitment if they met the following inclusion criteria: stable recurrent spontaneous angina episodes occurring ≥3 times per week despite ongoing anti-anginal therapy and angiographic evidence of the CSFP as indicated by TIMI-2 flow (i.e., requiring ≥3 beats to opacify a major epicardial vessel) in the absence of obstructive coronary artery disease. Exclusion criteria included the following: (i) an acute coronary syndrome admission within the past month; (ii) secondary causes of CSFP, such as the no-reflow phenomenon, clinically significant anaemia (haemoglobin < 100 g/L), uncontrolled atrial fibrillation (ventricular response rate > 108 bpm), and haemodynamically significant aortic stenosis (mean aortic valve gradient ≥ 50 mmHg); and (iii) contraindications to ticagrelor, including active pathological bleeding disorders and prior intolerance to ticagrelor.

### 2.3. Randomisation Protocol

Following an initial visit, when clinical history and informed consent were obtained, patients were asked to keep an angina diary for two weeks to assess the baseline frequency and characteristics of their angina. Participants who were experiencing angina at least 3 times/week were then randomised to either twice daily ticagrelor (90 mg) or matching placebo treatment using a computer-generated algorithm, with the sequence known only to the hospital clinical study pharmacist who had no contact with the patients. Participants received the initial phase of ticagrelor or placebo for a duration of four weeks. During this period, they were assessed for any adverse events via phone call at 1 week and at the end of the 4-week phase visit. Subsequently, patients commenced a two-week washout period and then crossed over to the alternative ticagrelor/placebo therapy for another four weeks, with the above study protocol repeated. Throughout this study, regular maintenance anti-anginal medications were maintained.

### 2.4. Study Endpoints

The primary endpoint was the total number of angina episodes recorded over each 4-week treatment period. Secondary endpoints were the frequency of prolonged angina episodes (lasting more than 20 min), the consumption of short-acting nitrates, and various indices from the SAQ, such as angina frequency, quality of life, physical limitation, and treatment satisfaction scores. Additionally, the SF-36 indices were used to assess physical and mental health summary scores. These instruments have been previously validated for patients with angina and provide important insights into the impact on health status [19,20]. Safety endpoints included frequency and severity of adverse events and frequency of bleeding events.

### 2.5. Data Acquisition

A comprehensive purpose-built case report form and angina diary were designed to capture detailed information on patient risk factors, past cardiac history, and current medications as well as angina symptoms. Participants were instructed to keep an angina diary throughout this study, including during the screening period (2 weeks), phase 1 (4 weeks), washout period (2 weeks), and phase 2 (4 weeks). This diary documented the frequency and severity of angina episodes, the duration of each episode (with prolonged episodes defined as lasting more than 20 min), and the consumption of glyceryl trinitrate (GTN).

During each visit, a cardiologist performed a medical assessment, which included monitoring pulse and blood pressure and reviewing the ECG taken during the visit. The angina diary was examined, and any changes in medication, occurrences of adverse reactions or events, and new diagnoses were documented. In addition to these clinical measures, during the last week of each treatment phase, an electrocardiograph was performed, and the SF-36 and SAQ health status questionnaires were administered. Patient adherence to the prescribed medication regimen was recorded at the conclusion of each treatment phase through a pill count.

### 2.6. Statistical Methods

This study used a double-blind methodology, and all analyses were carried out with the study treatment assignments concealed. A linear mixed-effects model was utilised to evaluate the relationship between clinical variables (such as angina frequency and prolonged angina episodes) and fixed effects, including treatment group (placebo or ticagrelor), study arm order (placebo followed by ticagrelor or ticagrelor followed by placebo), and continuous week (spanning phase 1 and phase 2 weeks) in this crossover trial. Participant ID numbers were included as a random effect with a compound symmetry covariance structure to account for repeated measurements over time. Linear mixed-effects models were employed to examine the relationships between the SAQ and SF-36 outcome variables and the fixed effects of treatment group (placebo or ticagrelor) and study arm order (placebo followed by ticagrelor or ticagrelor followed by placebo). The normality of residuals and homoscedasticity were verified by inspecting histograms of residuals and scatter plots of predicted values against residuals. Data were analysed in a long format using SAS On Demand for Academics (SAS Institute Inc., 2021). A *p* value of <0.05 was considered statistically significant.

Total angina frequency served as the primary endpoint of this study, and the sample size was determined based on this measure. A study evaluating the anti-anginal effects of mibefradil on patients with symptomatic CSFP reported a mean of 28 ± 31 angina episodes per month during placebo therapy. This study demonstrated a 56% reduction in total angina frequency with the active drug [21]. To detect a 50% reduction in angina frequency with ticagrelor using a crossover design, it was calculated that 29 patients would be required to achieve 80% power with an alpha level of 0.05.

## 3. Results

### 3.1. Study Population and Clinical Characteristics

**Figure 2:**
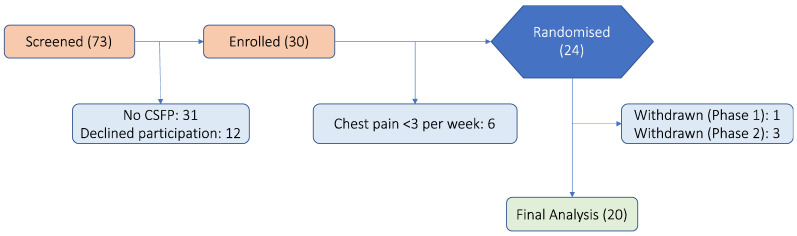
Consort diagram. This figure illustrates the patient flow from initial enrolment to final analysis. It details the number of patients assessed for eligibility, those randomised to either the ticagrelor or placebo group, the adherence and follow-up process, and the number of patients included in the final analysis after completing the study phases and washout period. CSFP = coronary slow flow phenomenon.

Between August 2016 and September 2021, 24 participants were randomised into this study with 4 patients subsequently withdrawing (Figure 2). The participants who completed the protocol experienced no major adverse effects, and medication compliance was over 95%, as determined by pill counts. The clinical characteristics of the 20 patients (40% women) included in the analyses are shown in Table 1. The recruited patients were more often male with mean age of 61.5 ± 10.5 years. All the participants were on at least one conventional anti-anginal treatment (Table 1), yet their baseline mean angina frequency was 6.3 (1.0) angina episodes/week.

### 3.2. Endpoints

**Figure 3:**
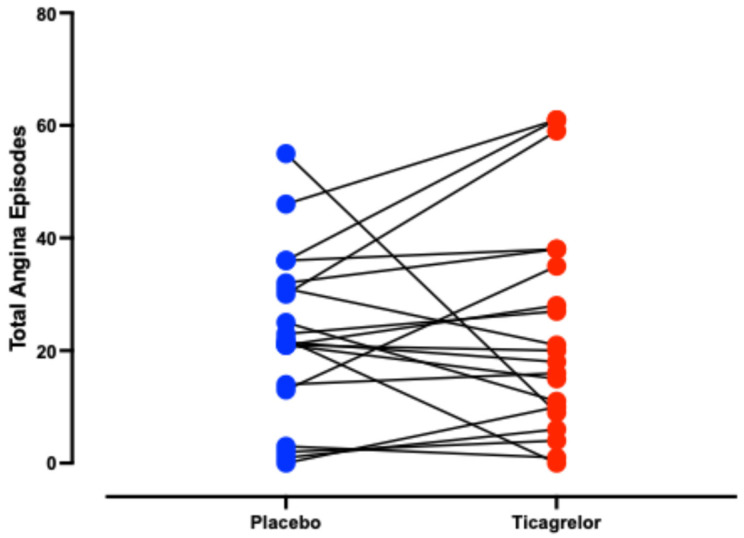
Total angina episodes by treatment group. Each point represents an individual patient, with data points colour-coded to reflect the patient’s treatment phase: blue for when they were on placebo, and red for when they were on ticagrelor. Statistical analysis revealed no significant difference between the two groups (*p* > 0.05).

The total angina frequency, which was the primary endpoint, did not differ significantly between the active and placebo treatments (Table 2), with a difference of just 2.2% observed between the two (Figure 3). Similarly, there was no significant difference in prolonged angina episodes, mean pain score severity, or nitrate consumption between treatments (Table 2). Both the SF-36 and SAQ indices revealed no significant improvement in physical or mental summary scores or in angina frequency with ticagrelor therapy (Table 3).

### 3.3. Safety Endpoints

The safety and tolerability analyses revealed that participants reported a total of 38 non-serious adverse events during the trial. Among these, 16 were associated with placebo treatment, while 22 were related to ticagrelor treatment. Only one participant had their regimen adjusted to every other day; no other participants’ regimens were changed due to non-serious adverse events. No serious adverse events were observed throughout the study.

## 4. Discussion

**Figure 4:**
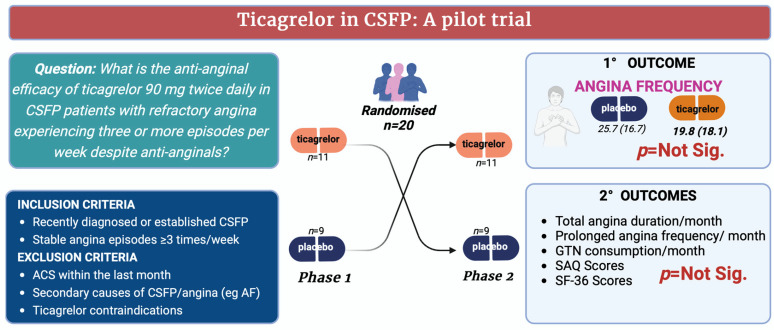
Central illustration depicts the study findings. It visually summarises the key outcomes and insights of this study. n = number, mg = milligrams, CFSP = coronary slow flow phenomenon, AF = atrial fibrillation, GTN = glyceryl trinitrate, SAQ = Seattle Angina Questionnaire, SF-36 = Short Form-36. Created with BioRender.com.

Our study investigated the anti-anginal efficacy of ticagrelor, a P2Y12 receptor antagonist that inhibits erythrocyte adenosine uptake thereby increasing plasma adenosine levels—a potent microvascular vasodilator [22]. Since the underlying pathophysiological mechanism of CSFP is microvascular constriction, ticagrelor would be expected to alleviate this constriction and thus reduce the frequency of spontaneous angina episodes in the 20 patients with refractory angina (Figure 3). However, this randomised controlled trial did not demonstrate any anti-anginal benefit in patients with refractory angina and CSFP (Figure 4). Several factors may have contributed to the lack of observed benefit.

The CSFP is a specific coronary microvascular disorder endotype, which involves an increased resting coronary vasomotor resistance, as reflected by the slow passage of contrast during diagnostic coronary angiography. It differs both clinically [6] and pathophysiologically [15] to other coronary microvascular disorders, with patients frequently experiencing rest angina [6], and the coronary flow reserve (a marker of coronary microvascular dysfunction) is frequently preserved [16]. The mechanism responsible for the angina symptoms in these patients is not completely understood, but endothelin-1 is thought to have a key role considering that patients have increased endothelin levels; the angiographic phenomenon is produced by intracoronary endothelin administration in animal models, and an endothelin blocker has been shown to have anti-anginal benefits [23]. Although adenosine pre-treatment has been shown to inhibit endothelin-1-induced coronary microvascular constriction in an animal model [24], it is possible that it is less effective in patients with CSFP. Specifically, CSFP-related microvascular constriction might be unresponsive to adenosine due to its specific association with endothelin-1. Additionally, microvascular constriction may not be the primary mechanism of angina in these patients. Instead, co-existing vasospasm could be contributing to their symptoms. Despite this, other vasodilators have shown efficacy in alleviating their symptoms, suggesting that the primary anginal mechanism might involve factors beyond microvascular constriction alone. Selection bias could also be a factor, as this study may have included patients with recalcitrant refractory angina, representing the most severe cases and potentially skewing the results. Thus, ticagrelor may not be an effective anti-anginal in patients with CSFP but may be of benefit in other coronary microvascular disorder endotypes where patients have normal resting blood flow, effort-related angina, and/or an impaired coronary flow reserve. Hence, further studies are required to evaluate the efficacy of ticagrelor in these microvascular endotypes.

Another explanation that may account for the lack of effect in this study is the refractory angina experienced by the study patients. The recruited patients were all experiencing spontaneous angina episodes at least three times a week despite being on conventional anti-anginal therapy, although there is no established first-line therapy for patients with coronary microvascular disorders. It is conceivable that the response to ticagrelor could differ in treatment-naïve patients or those with less-advanced disease. Future research may benefit from investigating the drug’s efficacy in de novo CSFP cases. In addition, age may have affected the observed responses due to age-related pharmacokinetic differences [25]. Ticagrelor levels are higher in elderly patients, although resting plasma adenosine levels are not significantly altered by age. The study cohort was predominantly middle-aged (mean age 61 ± 10 years), with only two patients being elderly (over 75 years of age). The low prevalence of elderly patients in this study limits the ability to generalise the findings to an older population.

The alternative explanation for the lack of response to ticagrelor in this study is that the drug itself has limited anti-anginal benefits. Ticagrelor may not be a potent vasodilator, which limits its ability to significantly alleviate angina symptoms, even though it can improve CFR. Additionally, the dose of ticagrelor used might have been suboptimal, which could have affected its efficacy. Moreover, the evidence for ticagrelor might not fully address its effectiveness in chronic versus acute settings, which could also impact its overall benefit in treating refractory angina secondary to the CSFP. Although the sample size was small and an argument can be made that this study was too under-powered to see a beneficial effect, it is noteworthy that the same study design and sample size using mibefradil (a novel calcium channel blocker) demonstrated a clear benefit of the latter agent in patients with the CSFP [21]. Thus, at least in relation to patients with refractory angina secondary to the CSFP, mibefradil is a more effective anti-anginal agent compared with ticagrelor.

### 4.1. Study Limitation

This study did not achieve its intended recruitment target and thus could be considered under-powered. Study recruitment was impaired by the COVID-19 pandemic, and this study was terminated since the study drug (ticagrelor/placebo preparation) had expired and further supplies were unavailable. Despite not achieving the recruitment target, as above, compared to mibefradil, ticagrelor does not appear to be as effective. Furthermore, futility analysis based on the observed difference among the 20 recruited participants suggests that it would necessitate a total of 35 patients (15 additional patients) to detect a 50% difference in angina frequency between the two treatments.

### 4.2. Gaps in Evidence and Future Directions

Ticagrelor, by inhibiting P2Y12 receptor activation, blocking platelet glycoprotein IIb/IIIa, and binding to fibrinogen, has a rapid onset and consistent antiplatelet effect, with well-established efficacy in acute coronary syndromes [26]. Clinical trials investigating ticagrelor’s effect on coronary microvascular function have produced mixed results. However, a systematic review and meta-analysis [27] of 16 randomised controlled trials, including 3676 patients with acute coronary syndrome who underwent percutaneous coronary intervention (PCI), found that ticagrelor significantly improved coronary microvascular function. Specifically, ticagrelor was associated with increased coronary flow reserve, myocardial blush grade, and TIMI flow grade, and a reduction in the index of microvascular resistance (IMR) and corrected thrombolysis in myocardial infarction frame count (cTFC). These findings align with evidence from large clinical trials, indicating that ticagrelor reduces the incidence of CMD in patients with acute coronary syndromes. However, the impact of ticagrelor on coronary microvascular function in chronic conditions such as CSFP remains underexplored. While the current trial was unable to demonstrate a significant benefit, this was likely due to its smaller sample size. Given the distinct pathophysiological mechanisms of CSFP compared to acute coronary syndromes, further research is needed. An adequately powered study could provide insights into whether ticagrelor’s antiplatelet and potential pleiotropic effects extend to improving coronary microvascular function in CSFP. Future research should focus on evaluating the long-term effects of ticagrelor on coronary flow in CSFP, understanding the mechanisms behind any potential benefits, and comparing ticagrelor with other therapies targeting chronic microvascular dysfunction. Addressing these gaps through comprehensive clinical trials will be crucial for determining ticagrelor’s role in managing CSFP and enhancing outcomes for patients with this chronic condition.

## 5. Conclusions

In conclusion, this study did not demonstrate any significant anti-anginal benefit of ticagrelor 90 mg taken twice daily in patients with the CSFP and spontaneous angina episodes unresponsive to conventional anti-anginal agents.

## Figures and Tables

**Table 1:** Baseline clinical characteristics of patients prior to randomisation.

Participant Characteristics	
Number of patients	20
Age (m, SD)	61.5 ± 10.5 years
Female %	40% (8)
Smoking (current) %	0
Hypertension (on treatment) %	50% (10)
Diabetes (on treatment) %	15% (2)
Family of CAD %	45% (9)
Hypercholesterolaemia (on treatment)	55% (11)
Height (m, SD)	170 ± 9.2 cm
Weight (m, SD)	83.1 ± 17.8 kg
Maintenance medications	
Aspirin %	50% (10)
Statins %	75% (15)
Calcium channel blockers %	75% (15)
ACE-inhibitors/ARB %	25% (5)
Beta blockers %	10% (2)
Nitrates %	70% (14)
Angina diary	
Angina episodes/week (m, SE)	6.3 (1.0)
Total duration/week (min, SE)	393.5 (162.5)
GTN consumption/week (m, SE)	2.1 (0.9)
SAQ components	
Angina frequency (m, SE)	50.5 (3.2)
Physical limitation (m, SE)	78.6 (5.2)
Treatment satisfaction (m, SE)	85.5 (2.5)
Angina-specific quality of life (m, SE)	60.8 (4.4)
SF-36 components	
Physical Component Summary (m, SE)	41.1 (3.0)
Mental Component Summary (m, SE)	51.5 (2.1)

This table summarises the baseline clinical characteristics of the patients before they were randomised. The data include demographic information, medical history, and relevant clinical measurements. CAD (coronary artery disease), ACE (angiotensin-converting enzyme), ARB (angiotensin II receptor blockers), GTN (glyceryl trinitrate), SAQ (Seattle Angina Questionnaire), SF-36 (Short Form 36 Health Survey). m = mean number; SD = standard deviation, SE = standard error; % = percentage

**Table 2:** Angina diary endpoints comparing placebo and ticagrelor groups.

	Placebo Mean (SD)	Ticagrelor Mean (SD)	Mean Difference, Mean Ratio or Incidence Rate Ratio (95% CI)	*p*
Total angina frequency/month	25.7 (16.7)	19.8 (18.1)	−6.5 (−21.2, 8.3)	0.3678
Total angina duration [min]/month	1009.0 (1565.0)	691.7 (1302.7)	0.17 (0.01, 2.61)	0.1897
Mean angina episode duration [min]/month	172.0 (289.1)	99.5 (133.1)	0.26 (0.03, 2.57)	0.2310
Mean angina episode severity/month	12.3 (5.9)	8.4 (6.3)	−4.01 (−9.14, 1.12)	0.1182
Prolonged angina frequency/month	11.3 (16.5)	7.7 (13.2)	0.68 (0.20, 2.35)	0.5428
Mean nitrate consumption/month	9.2 (14.3)	4.0 (8.2)	0.43 (0.10, 1.97)	0.2785

Comparison of various angina-related endpoints between placebo and ticagrelor groups. Results are presented as mean (SD), mean difference, mean ratio, or incidence rate ratio (95% CI) with corresponding *p*-values. SD = standard deviation, % = percentage, CI = confidence interval, p = probability value, min = minutes.

**Table 3:** Health status instruments.

Outcome	Placebo Mean (SE)	Ticagrelor Mean (SE)	∆ (95% CI)	*p*
***SAQ (score 0–100; recorded at the end of each phase)***				
Angina frequency	59.2 (5.0)	59.9 (5.0)	−0.71 (−13.10, 11.69)	0.9059
Physical limitation	78.7 (5.4)	76.3 (5.4)	2.46 (−6.69, 11.61)	0.5788
Treatment satisfaction	86.1 (3.1)	88.1 (3.1)	−1.96 (−8.33, 4.41)	0.5267
Quality of life	57.5 (5.8)	65.2 (5.8)	−7.70 (−20.47, 5.07)	0.2213
***SF-36 Generic HRQoL Questionnaire (score 0–100; recorded at the end of each phase)***				
SF-36-Physical Component Summary	44.3 (2.6)	42.9 (2.6)	1.40 (−1.84, 4.64)	0.3752
SF-36 Mental Component Summary	50.4 (2.1)	50.6 (2.1)	−0.19 (−3.07, 2.70)	0.8929

This table presents the mean scores (±SE) for various health status instruments recorded at the end of each phase of this study for both the placebo and ticagrelor groups. This table includes outcomes from the Seattle Angina Questionnaire (SAQ) and the SF-36 (Short-Form 36) Generic HRQoL Questionnaire. SE = standard error, % = percentage, CI = confidence interval, p = probability value.

## Data Availability

The data presented in this study are available on request from the corresponding author. The data are not publicly available due to privacy and ethical restrictions.

## Clinical Trial Registration

ACTRN12616000388415.
